# E7080, a multi-targeted tyrosine kinase inhibitor suppresses tumor cell migration and invasion

**DOI:** 10.1186/1471-2407-11-309

**Published:** 2011-07-22

**Authors:** Hilary Glen, Susan Mason, Hitesh Patel, Kenneth Macleod, Valerie G Brunton

**Affiliations:** 1 Beatson Institute for Cancer Research, Glasgow, G61, 1BD, UK; 2 Edinburgh Cancer Research Centre, University of Edinburgh, Edinburgh, EH4 2XR, UK

## Abstract

**Background:**

E7080 is an orally active multi-targeted kinase inhibitor whose targets include vascular endothelial growth factor receptors (VEGFR), fibroblast growth factor receptor (FGFR) and platelet derived growth factor receptors (PDGFR). It has been shown to inhibit tumor angiogenesis by targeting endothelial cells. A number of the targets of E7080 are also expressed on tumor cells and here we have looked at the direct effects of E7080 on tumor cell behavior.

**Methods:**

Using a panel of human tumor cell lines we determined the effect of E7080 on cell proliferation, migration and invasion. Inhibition of FGFR and PDGFR signaling in the cells was measured.

**Results:**

E7080 had little effect on tumor cell proliferation. However, it blocked migration and invasion at concentrations that inhibited FGFR and PDGFR signaling. Knock-down of PDGFR-β in U2OS osteosarcoma cells also inhibited cell migration which, could not be further inhibited in the presence of E7080. Furthermore, E7080 could not inhibit the migration of a PDGFR negative cell line.

**Conclusion:**

E7080 does not significantly affect tumor cell proliferation but can inhibit their migration and invasion at concentrations that both inhibit its known targets and are achievable clinically.

## Background

Angiogenesis, the formation of new blood vessels, is required for tumor growth and metastasis [1,2]. The ability of tumors to promote angiogenesis is driven by expression of pro-angiogenic factors such as VEGF, bFGF, PDGF and transforming growth factor-β (TGF-β) of which VEGF is the most important [1,3]. VEGF exerts its actions through two receptor tyrosine kinases, VEGFR-1 and VEGFR-2, but it is signaling via VEGFR-2 that is important for tumor angiogenesis [4]. A third VEGF receptor VEGFR-3, is important for lymphangiogenesis and is activated by two different ligands, VEGF-C and VEGF-D [5]. In view of the dependence of tumors on angiogenesis for sustained growth, targeting angiogenesis has been the focus of much research into new anti-cancer therapies in recent years [6]. Direct angiogenesis inhibitors target endothelial cells by inhibiting their ability to proliferate, migrate and form new blood vessels. The first example of this was the use of an antibody to VEGF, which was able to inhibit the growth of tumors in mouse models [7]. Subsequently a humanized version of this antibody, bevacizumab, was developed which showed promising evidence of efficacy in pre-clinical models. However, this was not translated into the clinical setting. With the exception of renal carcinoma and glioblastoma multiforme [8,9], bevacizumab appears to have little activity as a single agent, although it does confer significant benefit when combined with cytotoxic agents and is now approved by the US Food and Drug Administration for use in colorectal, breast, lung and renal cancer, and glioblastoma multiforme. Other approaches to target angiogenesis have centered on the use of small molecule kinase inhibitors, which target VEGFR-2. Many of these inhibitors are multi-targeted with additional activity against one or a number of other receptor tyrosine kinases. One such inhibitor is E7080, an orally active multi-targeted tyrosine kinase inhibitor, which is currently in clinical development. Three dose escalation phase I trials of E7080 have now been performed, examining different dosing schedules [10-12]. In view of promising anti-tumor effects observed, a number of disease specific phase II and III trials, including melanoma, renal, thyroid, ovarian, hepatocellular and endometrial cancer, are now underway. E7080 is a potent inhibitor of VEGFR-2 and VEGFR-3 with IC_50_s of 4 and 5.2 nM respectively, but also has activity against VEGFR-1, FGFR-1, and PDGFRα/β tyrosine kinases although the IC_50_s are around 10 fold higher [13]. E7080 shows anti-tumor activity in human cancer cell xenografts, which has been attributed to its ability to inhibit angiogenesis predominantly through effects on VEGFR-2 inhibition but also through inhibition of KIT and FGFR-1 [13-15]. Inhibition of VEGFR-3 mediated lymphangiogenesis by E7080 also contributed to its ability to suppress lymph node and lung metastases in a mammary tumor model [16]. Although these effects were not mediated via direct effects of E7080 on tumor cells two of the main targets of E7080, FGFR-1 and PDFGR, are expressed in a number of solid tumors. We therefore set out to determine whether E7080, in addition to effects on angiogenesis, could directly affect the behavior of epithelial tumor cells.

*FGFR-1 *amplification and over expression has been reported in a sub-set of breast cancers [17], lung squamous cell carcinoma [18] and also in oral squamous carcinoma, rhabodomyosarcoma, ovarian and bladder cancer [19]. In melanoma there is evidence for a bFGF/FGFR-1 autocrine loop driving proliferation *in vivo *[20,21] indicating that in some tumor types inhibitors such as E7080 may also have direct effects on the proliferation of tumor cells. The PDGFR and its ligands are also expressed in a number of different tumor types including gliomas, breast and ovarian cancer and there is evidence for PDGFR autocrine growth control in gliomas [22,23]. However, PDGFR signaling is also important in tumor stromal cells and many effects of PDGF in tumor cells may be mediated via paracrine activation of stromal cells [24,25] and in particular endothelial cells [23,26]. The effects of E7080 on tumor angiogenesis may therefore in part be mediated via inhibition of PDGFR signaling in endothelial cells. A number of small molecule tyrosine kinase inhibitors that target both the FGFR and the PDGFR are now in clinical development, although, like E7080, they also inhibit a number of other receptor tyrosine kinases making it difficult to determine the importance of targeting each individual receptor kinase to tumor development [19,22,27].

Cancer cell proliferation is often regarded as the most important aspect of cancer progression, and traditionally, anti-cancer treatment has focused on preventing growth of the tumor. However, other key aspects play a crucial role in cancer progression, including tissue invasion and metastasis [28]. A malignant tumor's ability to invade and migrate differentiates it from a benign tumor, and hence these processes are crucial for cancer progression. 90% of human cancer deaths are caused by distant metastases and therefore preventing cancer spread could have a huge impact on patient survival. Signaling via both the FGFR-1 and PDGFR has been associated with increased migration, invasion and metastatic potential [29-33].

We therefore set out to determine whether E7080 has any direct effects on tumor cell proliferation, migration and invasion. We show that although there was very little effect of E7080 on the proliferation of a range of human tumor cell lines both their migration and invasion could be blocked at concentrations of E7080 that inhibited signaling through FGFR-1 and PDGFR-β. Furthermore, we show that U2OS osteosarcoma cell migration was inhibited by both E7080 and knock-down of PDGFR-β expression suggesting that E7080 may directly affect the migratory capacity of tumor cells by targeting PDGFR-β. The ability of E7080 to inhibit both migration and invasion *in vitro *implies that it could potentially have significant benefit in the prevention (or at least in the reduction) of development of tumor metastases *in vivo*.

## Methods

### Cell culture and reagents

A375, U2OS, DU145 and human umbilical vein endothelial cells (HUVECs) were obtained from the American Type Culture Collection (Manassas, VA, USA). KM12C cells were from I Fidler (MD Anderson Cancer Centre, Houston, TX, USA) and DX3 and SK23 cells were a kind gift from B Ozanne (Beatson Institute, Glasgow, UK). DU145 and KM12C cells were grown in RPMI-1640 supplemented with 10% fetal bovine serum (FBS) and 2 mM glutamine and MEM supplemented with 10% FBS and 2 mM glutamine, 1 mM sodium pyruvate, 0.1 mM non-essential amino acids respectively. HUVECs were grown in media 199 supplemented with 20% FBS, heparin, 1% penicillin/streptomycin/fungizone and endothelial cell growth supplement. All other cell lines were grown in DMEM supplemented with 10% FBS and 2 mM glutamine. FBS was obtained from Autogen Bioclear (Calne, UK) and all other tissue culture reagents from Invitrogen (Paisley, UK). All lines were maintained in a humidified atmosphere of 5% CO_2 _at 37°C and were serum starved prior to growth factor stimulation. Growth factor treatments were: bFGF (100 ng/ml, 15 min), PDGF-BB (50 ng/ml, 15 min) and VEGF-A (100 ng/ml, 15 min)(all Upstate Biotechnology, Waltham, MA, USA). E7080 (Eisai Co. Ltd., Ibaraki, Japan) was prepared as a 10 mM stock in DMSO and diluted in culture media prior to use. For PDGFR-β knock-down experiments cells were transfected with either 50 nM of ON-TARGETplus SMARTpool PDGFR-β or non-targeting siRNA oligonucleotides according to the manufacturers guidelines (Dharmacon, Thermo Scientific, Loughborough, UK). 72 hours after transfection cell extracts were prepared or cells were plated out for wound healing assays as described below.

### Cell proliferation assay

3-(4,5-dimethylthiazol-2-yl)-2,5-diphenyltetrazolium bromide (MTT) proliferation assays were carried out as described previously [34]. Cells were treated with a range of E7080 concentrations for 72 hours. Curve fitting and generation of IC_50 _values was carried out using GraphPad Prism 4 software from quadruplicate wells.

### Immunoprecipitation and immunoblot analysis

Cell extracts were prepared as described previously [34] and then immunoprecipitated with PDGFR-β (Upstate Biotechnology), FRS2 (Santa Cruz, Santa Cruz, CA, USA) or VEGFR-2 (Cell Signaling, Danvers, MA, USA) antibodies overnight. Immune complexes were collected with protein-A or protein-G sepharose beads (Sigma, Gillingham, UK) and immunoblot analysis then carried out as described previously [34] using the above antibodies and also an anti-phosphotyrosine antibody (PY20, BD Transduction Laboratories, Oxford, UK).

### Migration assays

Cells were plated in 6 well plates at low cell density. After 24 hours E7080 was added and images captured every 5 minutes for 16 hours on a Nikon TE2000 microscope using a 10× objective. Image J software was used to track the cell nuclei of 20 separate cells per condition over time and calculate the total accumulated distance, euclidean distance (straight line distance travelled) and velocity. Wound healing assays were carried out on confluent monolayers of cells after 'wounds' were made by scoring the monolayer with a fine pipette tip. E7080 was then added and images captured every 15 minutes for 16 hours on a Nikon TE2000 microscope using a 10× objective. Wound closure was then quantified using Image J by calculating the distance between the wound edges from 8 images per treatment condition.

### Invasion assay

Inverse invasion assays were performed as described previously using FBS as a chemoattractant [35]. In brief, cells were seeded on the bottom of Transwell inserts (Corning, Fisher Scientific, Loughborough, UK) containing polymerized collagen type I (Becton Dickinson, Oxford, UK). Transwell inserts were then placed in serum-free medium and medium supplemented with 10% FBS was placed on top of the matrix in the presence or absence of E7080. Five days after seeding invading cells were stained with Calcein AM (Invitrogen) and visualized using a Leica SP2 confocal microscope. Serial optical sections were captured at 15 μm intervals and quantified using ImageJ analysis software. Invasion was calculated as cells that had moved more than 20 μm into the collagen.

## Results

### Effects of E7080 on tumor cell proliferation

**Figure 1:**
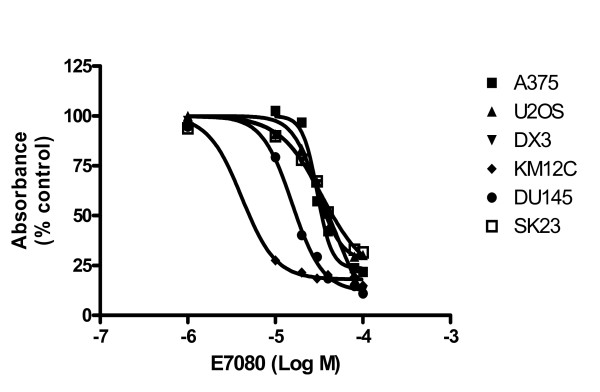
**Effect of E7080 on tumor cell proliferation**. Dose-response curves for E7080 in a panel of cell lines. Results are shown from representative experiments for each cell line in a series of at least 3 where values are the mean of 4 replicates.

Initially we chose a panel of 6 human cell lines representing a number of different tumor types and carried out MTT assays to determine the effect of E7080 on their proliferation. Dose-response curves for E7080 are shown in Figure 1 from which the IC_50 _value for E7080 in each cell line was calculated (Table 1). In the majority of the cell lines, E7080 only inhibited proliferation at high concentrations (mean IC_50_s 23.6 - 44.17 μM) while the IC_50 _in the KM12C colon cancer cell line was 9.54 μM.

**Table 1:** IC_50 _values for E7080 in a panel of cell lines

Cell line	Origin	**IC**_**50**_**(μM)**^**a**^	95% Confidence interval
A375	Melanoma	44.17	37.24 - 51.09

DU145	Prostate cancer	23.62	20.02 - 27.20

DX3	Melanoma	33.95	21.11 - 46.79

KM12C	Colon cancer	9.54	7.94 - 11.14

SK23	Melanoma	42.57	40.19 - 44.94

U2OS	Osteosarcoma	34.82	31.36 - 38.28

^a^Mean values from at least 3 experiments

### Inhibition of FGFR-1 and PDGFR-β signaling by E7080

**Figure 2:**
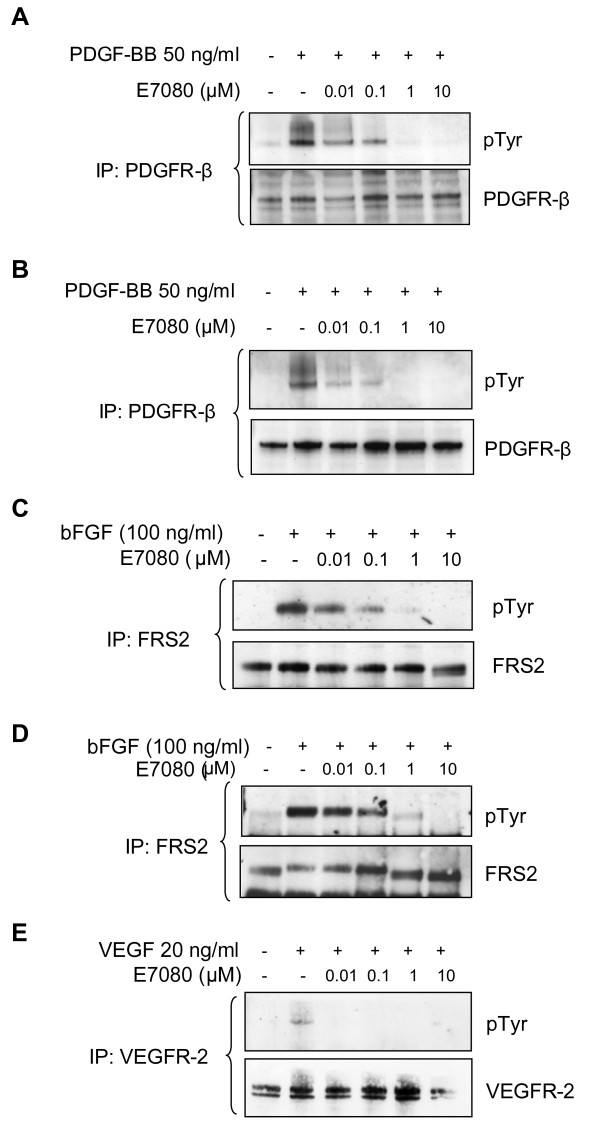
**Inhibition of PDGFR-β and FGFR-1 signaling by E7080**. (A) DX3 and (B) U2OS cells were treated with increasing concentrations of E7080, then stimulated with PDGF-BB. Lysates were prepared and the PDGFR-β immunoprecipitated and the immune complexes resolved by SDS-PAGE and probed with an anti-phosphotyrosine (pTyr) antibody. Membranes were then stripped and reprobed with an anti-PDGFR-β antibody. (C) DX3 and (D) U2OS cells were treated with increasing concentrations of E7080, then stimulated with bFGF. Lysates were prepared and FRS2 immunoprecipitated and the immune complexes then resolved by SDS-PAGE and probed with an anti-pTyr antibody. Membranes were then stripped and reprobed with an anti-FRS2 antibody. (E) HUVECs were treated with increasing concentrations of E7080, then stimulated with VEGF-A. Lysates were prepared and the VEGFR-2 immunoprecipitated and the immune complexes then resolved by SDS-PAGE and probed with an anti-pTyr antibody. Membranes were then stripped and reprobed with an anti-VEGFR-2 antibody.

To establish the concentration of E7080 required to inhibit activation and downstream signaling from FGFR-1 and PDGFR-β we chose two cell lines which expressed both receptors: DX3 melanoma and U2OS osteosarcoma. PDGFR-β autophosphorylation in response to stimulation with its ligand PDGF-BB was used as a measure of PDGFR-β activation. PDGFR-β phosphorylation was seen in both DX3 and U2OS cells following stimulation with PDGF-BB (Figures 2A and 2B). Treatment with E7080 resulted in a dose-dependent inhibition of PDGFR-β phosphorylation in both cell lines with concentrations of 1 μM and above resulting in complete inhibition (Figures 2A and 2B). Direct measurement of FGFR-1 autophosphorylation was not possible due to the lack of FGFR-1 antibodies for western blotting and immunoprecipitation. The signaling cascade downstream of FGFR-1 activation by its ligand involves tyrosine phosphorylation of an adaptor protein FRS2 followed by the recruitment of several signaling molecules and subsequent activation of pathways such as the MAPK signaling pathway. FRS2 phosphorylation is FGFR-1 dependent and acts as a read out of FGFR-1 activity in the cells. DX3 and U2OS cells were treated with increasing concentrations of E7080 and then stimulated with bFGF. A marked increase in FRS2 phosphorylation was observed in DX3 cells following bFGF stimulation (Figure 2C). There was a dose dependent inhibition of bFGF stimulated FRS2 phosphorylation by E7080 with complete inhibition seen at concentrations of 1 μM and above (Figure 2C). There was also a dose dependent inhibition of bFGF-induced phosphorylation of FRS2 in U2OS cells with again concentrations of 1 μM resulting in complete inhibition (Figure 2D). For comparison we also looked at the ability of E7080 to inhibit VEGF-A induced activation of VEGFR-2 in HUVECs. Treatment with VEGF-A resulted in phosphorylation of VEGFR-2, which was inhibited by pre-treatment with E7080 at concentrations as low as 0.01 μM (Figure 2E).

### E7080 inhibits cell migration and invasion

**Figure 3:**
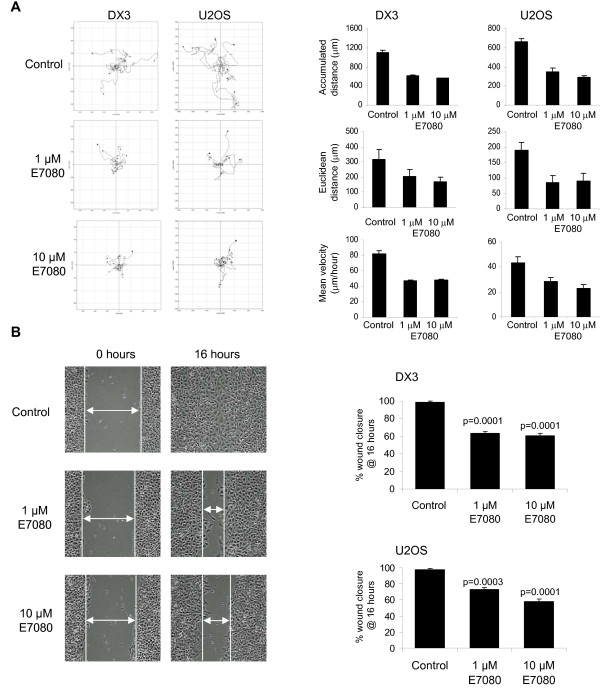
**Inhibition of cell migration by E7080**. (A) Individual DX3 and U2OS cell movements were captured over 16 hours in the presence or absence of E7080 and then tracked and plotted using Image J. Representative plots show 8 cells for each condition. (B) Accumulated and euclidean distances travelled and velocity were quantified from at least 20 cells per condition and plotted as mean + s.e.m., p values, comparing treated cells with controls, all <0.01, Paired t-test. (C) Representative images (x10) of wound healing assay in U2OS cells. Cells were grown to confluency, then wounded and wound closure in the presence or absence of E7080 followed over 16 hours. (D) % wound closure was calculated after 16 hours from 8 images. Values are mean + s.e.m., p values comparing treated cells with controls were calculated using Paired t-test.

**Figure 4:**
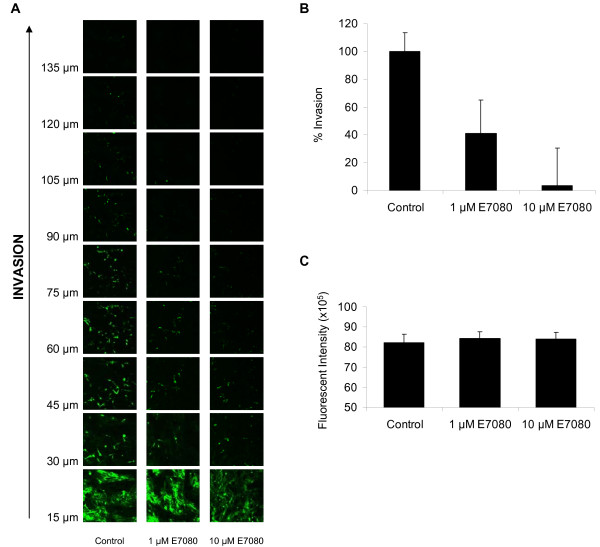
**Inhibition of DX3 cell invasion by E7080**. (A) Invasion of DX3 cells into Matrigel in the presence or absence of E7080 was measured: DX3 cells were stained with calcien AM and images taken every 15 μm. (B) Quantification of invasion is shown for a representative experiment in a series of three. Values are mean + s.e.m. of two transwells with 4 fields per transwell. p values comparing treated cells with controls were calculated using Paired t-test. (C) The fluorescent intensity of DX3 cells on the upper side of the Transwell was measured in untreated and E7080 treated cells following staining with calcein AM. Results are mean + s.e.m from a representative experiment in a series of three.

Initially the effect of E7080 on the random migration of DX3 and U2OS cells was measured using time lapse videomicroscopy. E7080 treatment (both at 1 μM and 10 μM) resulted in a significant inhibition of both accumulative (total) and euclidean (total straight line) distance moved and this was also reflected in a reduction in the mean velocity of both cell lines (Figure 3A and 3B). To assess the effects of E7080 on directional cell migration wound healing assays were performed. In the presence of E7080 (both at 1 μM and 10 μM) the ability of both cell lines to move into the wounded area was significantly impaired. Importantly inhibition of cell migration was seen with concentrations as low as 1 μM E7080, which correlated with the concentration required to inhibit FGFR-1 and PDGFR signaling in these cells (Figure 2). Complete inhibition of cell migration was not seen in either cell lines indicating that other pathways that are not targeted by E7080 are also involved in their migration. E7080 also inhibited the ability of DX3 cells to invade into a Matrigel plug. Compared to untreated cells, invasion was significantly reduced to 45% by 1 μM E7080 and to 13% by 10 μM E7080 (Figure 4A and 4B). The reduction in invasion after treatment with E7080 was not due to an inability of cells to migrate across the Transwell filter, as there was no difference in the number of cells on the top of the filter in the presence of E7080 (Figure 4C).

### PDGFR-β signaling is required for U2OS cell migration

**Figure 5:**
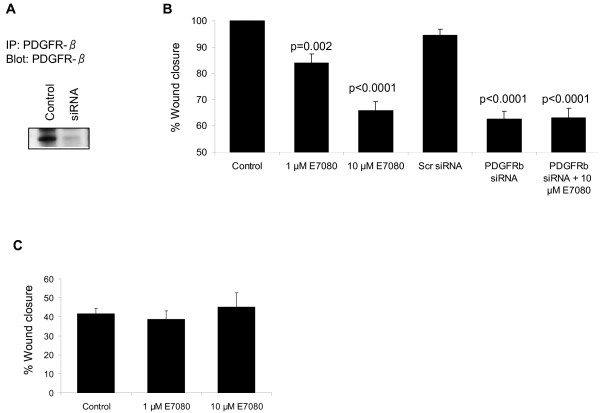
**PDGFR-β is required for U2OS cell migration**. (A) Cell lysates were prepared following transfection of U2OS cells with PDGFR-β or non-targeting siRNA oligonucleotides and PDGFR-β immunoprecipitated. The immune complexes were resolved by SDS-PAGE and probed with an anti-PDGFR-β antibody. (B) Wound healing assays with U2OS cells were carried out in the presence or absence of E7080 and/or PDGFR-β siRNA oligonucleotides. Values are mean + s.e.m. from a representative experiment in a series of three, p values comparing all with control were calculated using Paired t-test. (C) Wound healing assay in DU145 cells treated with E7080. Values are mean + s.e.m. from a representative experiment in a series of three.

As there is a large body of evidence linking PDGF receptor signaling with cell migration [36] we next set out to determine whether targeting PDGFR-β signaling could inhibit U2OS cell migration. PDGFR-β expression was knocked down in U2OS cells by siRNA (Figure 5A). Knock-down of PDGFR-β by siRNA resulted in a significant inhibition of cell migration while treatment with scrambled siRNA sequences had no effect (Figure 5B). Treatment with 10 μM E7080 resulted in a similar reduction in wound closure as PDGFR-β siRNA (35% versus 38% reduction respectively, p = 0.57). Treatment of U2OS cells with both E7080 and PDGFR-β siRNA did not result in a further inhibition of cell migration as compared to either treatment alone (Figure 5B), suggesting that the ability of E7080 to inhibit migration is mediated via inhibition of PDGFR-β signaling. To further explore whether the effect of E7080 on cell migration was mediated via inhibition of PDGFR-β, wound healing assays were performed using the human prostate cancer cell line DU145 that does not express PDGFR-β protein [37,38]. In the presence of both 1 μM and 10 μM E7080 no inhibition of DU145 migration was observed (Figure 5C).

## Discussion

E7080 is a promising new multi-targeted kinase inhibitor that is currently in clinical development. The main targets of E7080 are VEGFR-2 and VEGFR-3 and the preclinical work to date has focused on the anti-angiogenic activity of E7080. However, in this study we show that E7080 also has direct effects on tumor cells at concentrations that correlate with inhibition of other known targets, notably FGFR-1 and PDGFR-β. E7080 was able to inhibit both tumor cell migration and invasion but had little effect on tumor cell proliferation. As with many molecularly targeted agents there still remain questions about the clinical utility of E7080 in terms of which patients will benefit and in which setting treatment with E7080 would be most appropriate. A detailed preclinical investigation of the mechanism of action of drugs such as E7080, which have multiple kinase targets, is therefore essential to maximize its potential clinical application. FGFR-1 and PDGFR-β are both widely expressed in epithelial tumors and there is growing interest in both of these kinases as potential targets although many of the inhibitors currently available, as with E7080, also inhibit a number of other kinases [19,22,27]. It is likely therefore that such inhibitors will have multiple effects on both tumor cells and associated endothelial and stromal cells.

The pharmacokinetic analyses of patient samples from the phase I clinical trial of E7080 indicated that patients on the higher dose cohorts (receiving doses that will be recommended for further clinical development) typically achieved peak plasma concentrations of greater than 1 μM (manuscript submitted). This would suggest that inhibition of FGFR and PDGFR-β should be occurring in patients receiving treatment doses of E7080 and is greatly in excess of the concentrations required to inhibit VEGFR-2 activity (Figure 2). Inhibition of tumor cell proliferation was only seen at very high concentrations of E7080. These were in excess both of those achieved clinically and those required to inhibit the main known targets of E7080. This is in agreement with previous studies, which have shown minimal effects of E7080 on tumor cell growth *in vitro *[13]. However, the situation *in vivo *may be very different as the tumor micro-environment often plays an important part in tumor growth. For example, there is evidence that melanoma is driven in part by bFGF acting on FGFR-1 on tumor cells. bFGF is released from tumor cells, endothelial cells and surrounding stromal cells, and therefore acts in an autocrine and paracrine fashion. This may lead to high local concentrations of bFGF, which may then be important for driving tumor cell proliferation. In addition over-expression of receptors or activating mutations may mean that some tumor cells are more dependent on these kinases for proliferation.

Although the evidence for direct effects of E7080 on tumor proliferation are not compelling we did see significant effects of E7080 on tumor cell migration and invasion at concentrations that both inhibit its known targets and are achievable clinically. Our data suggests that these effects of E7080 may be due to inhibition of PDGFR signaling. PDGFR signaling has been known for many years to regulate cell migration [36,39] via activation of a number of signaling pathways including small Rho GTPases, phosphatidylinositol (PI)3-kinase and phospholipase (PL)C-γ [36] and in tumor cells this has been linked to activation of PI3-kinase [29,40]. PDGF signaling also regulates the invasion of tumor cells [29,31,41]. In pancreatic cancer this is associated with PDGF induced phosphorylation of MUC1 a transmembrane glycoprotein involved in pancreatic tumor progression and metastasis [31]. PDGFR signaling has been implicated in the development of metastases in a variety of human tumors, including oesophageal cancer [42], colorectal cancer [24,25], pancreatic cancer [31], sarcoma [43] and non-small cell lung cancer [44]. In addition in a mouse model of mammary carcinogenesis autocrine PDGFR signaling promotes metastasis through regulation of an epithelial-to-mesenchymal transition (EMT) and treatment with the PDGFR inhibitor imatinib suppressed metastasis [30].

In addition to our reported effects of E7080 on cell migration and invasion, E7080 significantly reduced lymphatic vessel density in MDA-MB-231 mammary tumor xenografts, which corresponded to a complete inhibition of both lymph node metastasis and lung metastasis in E7080-treated mice, thought to be due to the effects of E7080 on VEGFR-3 activity [16]. Thus in addition to effects on angiogenesis E7080 may have additional effects on the development of metastases. Anti-invasive strategies are increasingly being investigated in patients with cancer. However, the clinical development of anti-invasive therapies is often hampered by their lack of direct anti-tumour effects and the difficulties involved with trial design and as yet it is not clear how they will be used in the clinical setting.

## Conclusions

E7080 does not significantly affect tumor cell proliferation but can inhibit their migration and invasion. Direct effects of multi-kinase inhibitors such as E7080 on tumor cells should be considered as they enter the clinic and based on these findings, and given the knowledge we have gained regarding the favorable efficacy and tolerability of E7080 (even after long term use), consideration should be made to the potential effects of E7080 on the development of metastatic disease.

## Competing interests

VGB and HG received funding from Eisai Co. Ltd. for this study. SM, KM and HP declare that they have no competing interests.

## Authors' contributions

HG contributed to the conception and design of the study and acquisition and analysis of data. SM, HP & KM contributed to the acquisition and analysis of data. VGB contributed to the conception and design of the study and wrote the manuscript. All authors have read and approved the final manuscript.

## Pre-publication history

The pre-publication history for this paper can be accessed here:

http://www.biomedcentral.com/1471-2407/11/309/prepub
